# Neuroprotective Effects of Ginsenoside Rg3 in Depressed Mice via Inhibition of the C1q Complement Pathway

**DOI:** 10.1111/cns.70646

**Published:** 2025-11-07

**Authors:** Daofeng Yang, Xinran Xu, Xuerui Zhuo, Hui Cai, HaoTian Wang, Hao Liu, Yiming Sun

**Affiliations:** ^1^ Pharmacy Department The First Affiliated Hospital of Bengbu Medical University Bengbu Anhui China; ^2^ School of Pharmacy Bengbu Medical University Bengbu Anhui China; ^3^ Institute of Critical Care The First Affiliated Hospital of Bengbu Medical University Bengbu Anhui China

**Keywords:** complement C1q, depression, ginsenoside Rg3, microglial cells, synaptic phagocytosis

## Abstract

**Aims:**

To explore the neuroprotective effect of Ginsenoside Rg3 in a mouse model of depression induced by chronic restraint, and to elaborate whether it exerts a protective effect by inhibiting the upregulation of complement factor C1q and related microglial cell‐mediated synaptic injury.

**Methods:**

The mouse chronic restraint model of depression was prepared from male C57BL/6 mice. The depression‐like behaviors of mice were detected by sugar water preference, forced swimming test and tail suspension test. The neuronal phenotypes of mice were detected by Nissl staining, HE and Golgi staining. The expression of complement‐related pathway proteins was detected by Western Blot. The corticosterone content in the peripheral blood of mice in each group was detected by corticosterone kit. Molecular simulation and MST were used to detect the binding of Rg3 and C1q. Establish a co‐culture model of microglia and neurons; corticosterone stimulation and Rg3 pre‐protection were given to detect the damaging effect of microglial activation on neurons and the protective effect of Rg3.

**Results:**

In vivo experiments show that as depression progresses, the levels of complement factor C1q significantly increase, which is similar to the manifestations observed in depressed patients. Moreover, microglial cells in the hippocampus of depressed mice are significantly activated, while the number of neuronal synapses is significantly reduced, displaying apoptotic features. Ginsenoside Rg3, a drug with neuroprotective effects, reduces complement C1q levels and inhibits microglial activation, thereby protecting neurons and improving depression‐like behaviors. In vitro experiments show that CORT can induce microglial cells to secrete complement factor C1q, which may trigger neuronal apoptosis. Ginsenoside Rg3 may protect HT22 neurons by regulating microglial activation, reducing the secretion of complement factors, and inhibiting neuronal apoptosis.

**Conclusion:**

These results suggest that inhibiting excessive microglial activation and complement factor levels could be a potential strategy for treating depression.

## Graphical abstract

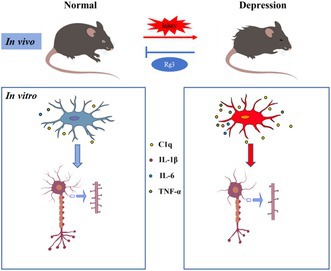

## 1 Introduction

Depression is a common mental disorder, mainly characterized by persistent low mood, slowed thinking, and reduced interest, often accompanied by cognitive impairments or physical symptoms. In severe cases, it may lead to suicidal thoughts and behaviors. According to statistics, approximately 350 million people globally suffer from depression, with 95 million sufferers in China, which is higher than the global average rate. By 2030, depression is expected to become one of the leading causes of global disease burden, causing enormous economic strain and severe harm to families and society [1, 2].

In recent years, the interaction between the immune system and the central nervous system (CNS) has played a crucial role in the pathophysiology of major depression. The innate immune system, as an ancient and primary defense mechanism, can rapidly recognize and respond to external pathogen invasions [3, 4]. Innate immune responses are primarily mediated by the complement system, which facilitates important immune responses between the innate and acquired immune systems and triggers various immune regulatory processes [5]. The activation of the complement system can occur through three pathways: the classical, lectin, and alternative pathways. Complement factor C1q, part of the classical pathway, acts as the initiating molecule and regulates various cellular functions, including cell differentiation, the occurrence of neurodegenerative diseases, cell macromolecule aggregation, and the clearance of apoptotic cell debris [6]. In addition to immune surveillance, C1q may also exhibit anti‐tumor effects and may play a role in the aging process. Under conditions of CNS inflammation, external damage, and cellular stress, complement proteins are expressed and secreted by neurons, astrocytes, and microglia [7, 8]. Further studies have shown that in the brains of mice from birth to adulthood, the major source of complement factor C1q is from microglia rather than peripheral blood or other CNS cells [9]. Studies have shown that abnormal activation of microglia in the occurrence of CNS diseases drives neuronal damage and synapse loss, a process belonging to the classical complement pathway. The abnormal increase of various complement factors in depression patients suggests that dysregulation of the complement pathway may play a role in the disease [10]. The role of the complement system in the onset and development of depression remains unclear. Therefore, exploring whether inhibiting the complement pathway can alleviate depression is one of the focuses of this study.

Ginsenosides, natural components of ginseng, have favorable pharmacological effects. The main studied types include Rb1, Rg1, Rg3, Re, Rd., and Rh1. Among them, ginsenoside Rg3 is a rare ginsenoside isolated from the precious Chinese herb ginseng and is one of its most effective components. It has various pharmacological effects with minimal toxicity and side effects, acting on the cardiovascular, endocrine, and immune systems to exhibit anti‐tumor, anti‐cardiovascular disease, anti‐oxidant, anti‐fatigue, and anti‐inflammatory effects [11, 12, 13]. Ginsenoside Rg3 acts on the CNS, providing protective effects for neural cells and showing potential as a treatment for neurological diseases [14, 15, 16]. Previous studies have mainly reported that Rg3 exerts antidepressant effects through pathways such as regulating the HPA axis, neurotrophic factor BDNF, monoamine neurotransmitters, antioxidant stress, and anti‐apoptosis. Targeting C1q‐mediated neuroinflammation and synaptic loss may be a previously underemphasized new mechanism by which Rg3 exerts neuroprotective effects. Hence, we hypothesize that ginsenoside Rg3 improves depressive‐like behaviors in mice by inhibiting the upregulation of C1q, reducing abnormal activation of microglia, and alleviating the loss of neuronal synapses.

## 2 Materials and Methods

### 2.1 Animals and Cell Lines

Male C57BL/6 mice, aged 8 weeks and weighing 22 ± 2 g were purchased from Hangzhou Ziyuan Laboratory Animal Technology Co. Ltd., with the animal production license number SCXK (Zhe) 2019–0004. The mice were housed under standard environmental conditions, with a temperature maintained at 22°C ± 2°C, humidity at 50%–60%, and provided with sufficient food and water, following the natural light–dark cycle. The mice started the experiment 1 week after acclimating to the environment. All experiments were conducted according to relevant guidelines, and the use of experimental animals was approved by the Animal Ethics Committee of Bengbu Medical College, animal Ethics No. 105 of 2021. The experimental procedures adhered to the guidelines for the use of laboratory animals.

BV2 microglial cells and HT22 neuronal cells were cultured in DMEM‐F12 and DMEM media, respectively. The culture media contained 10% fetal bovine serum and 1% penicillin–streptomycin. All cells were maintained in a 37°C incubator with 5% CO_2_.

### 2.2 Experimental Grouping

The mice were first divided into four groups: control group (CON group), ginsenoside Rg3 group, chronic restraint group (CRS group), and CRS + ginsenoside Rg3 group (CON+Rg3). The control group consisted of 8 mice, and the CRS model group consisted of 40 mice. The mice in the CRS model group were restrained for 8 h per day. After 8 weeks of continuous modeling, open field and sucrose preference behavioral tests were conducted. By comparing with the normal control group, the total movement distance and sucrose preference rate in the CRS model group were observed to see if there was a significant reduction, which would indicate the successful establishment of the depression model. Once the model was confirmed, based on the behavioral results, the CRS model mice were randomly divided into five groups: CRS model group, CRS + low‐dose ginsenoside Rg3 group (10 mg/kg), CRS + medium‐dose ginsenoside Rg3 group (20 mg/kg), CRS + high‐dose ginsenoside Rg3 group (40 mg/kg), and CRS + fluoxetine group (10 mg/kg). Each group contained 8 mice to ensure that there were no significant differences in the behavioral test results between the five groups, thereby ensuring the reliability and accuracy of subsequent experiments.

### 2.3 Depression Model in Mice and Administration Method

Mice were first allowed to acclimate to the environment for 1 week in the animal facility, with a 12 h light–dark cycle. During this period, they had normal access to food and water. The male C57BL/6 mice were then placed into 50 mL centrifuge tubes, which were modified by creating six ventilation holes. The mice were placed in the tubes, with their tails exposed to the air through the central hole in the cap. The mice were continuously restrained for 4 weeks, 8 h per day. During the restraint, they were not allowed to drink or eat, but were normally fed at other times [17, 18].

### 2.4 Behavioral Tests

#### 2.4.1 Sucrose Preference Test (SPT)

Two identical drinking bottles were used, one containing a 1% sucrose solution and the other containing pure water, and both bottles were weighed. Prior to the experiment, the two bottles were placed in the cage for the mice to adapt to the environment for 24 h. To prevent the mice from developing a preference for a specific drinking location, the positions of the two bottles were swapped during both the pre‐adaptation and formal testing phases. At the end of the experiment, the water bottles were weighed again. The difference in weight before and after the experiment was used to calculate the total consumption of pure water, sucrose solution, and the sucrose preference rate.

#### 2.4.2 Opening Field Test (OFT)

OFT was used to evaluate the spontaneous exploratory behavior and depression‐like behavior in mice. Mice were individually placed in the center of a box (50 cm × 50 cm × 45 cm) under dark conditions. Recorded the behavior of the mice for 6 min and analyze the time they stayed in the center of the box for 4 min.

#### 2.4.3 Forced Swimming Test (FST)

Prepare a large transparent glass beaker with a volume of 2 L, add approximately 1800 mL of double‐distilled water, and ensure that the mice can effectively suspend in the water. Record and time the swimming behavior of the mice within 6 min, and count the immobile time of the mice in the last 4 min.

#### 2.4.4 Tail Suspension Test (TST)

The tail of the mouse was fixed separately with tape at the last third of its length. The tail was suspended on the support pole at the top of the box (15 cm × 12.5 cm × 45 cm), with the mouse's head facing down. The behavior of each mouse for 6 min was recorded with a camera, and the time of immobility for 4 min after the experiment was measured.

### 2.5 Proteomics Experiment

Three mice were selected from both the normal group and the chronic restraint group, and their hippocampal brain tissues were extracted. The quality of the original sequencing data was examined using Multi quality control (QC), and low‐quality reads and connector sequences were removed using Cut adapt. clean reads were aligned to the mouse reference genome using hierarchical Indexing for Spliced Alignment of Transcripts 2 (HISAT2). The gene expression level was calculated by HT Seq‐count. The original expression level data were standardized using DESeq2, and significantly differentially expressed genes were screened based on Fold Change (FC). Through Kyoto encyclopedia of genes and genomes (KEGG) analysis, the changes of biological pathways in RNA sequencing data of mouse hippocampal tissue can be systematically understood.

### 2.6 Western Blot (WB)

According to the weight of the hippocampal tissue, add the lysis buffer at a ratio of 1:7. Put the hippocampal tissue into the homogenizer and grind it thoroughly. Set the program to 60 Hz for 60 s. The mixture was centrifuged at 12,000 rpm at 4°C for 20 min, the supernatant was collected, and protein quantification was performed. After quantification, the mixture was heated at 95°C for 5 min to denature the proteins. Run the gel at a constant voltage of 80 V for 30 min. After the sample reaches the separation gel, increase the voltage to 120 V and continue electrophoresis for 90 min. After electrophoresis, the protein was transferred onto the PVDF membrane. Incubate the membrane with the primary antibody at 4°C overnight. Then incubate with the secondary antibody at room temperature for 1 h. Exposure was performed using the Micro Chemi chemiluminescence imaging system, and the intensity of protein bands was analyzed and quantified using ImageJ software.

### 2.7 Golgi Staining

The brain tissue is placed into a 24‐well plate and 1 mL of mixed solutions A and B is added. After 2 weeks, solutions A and B are removed and replaced with solution C. After soaking for 5 days, the tissue is sectioned and the slices are transferred onto microscope slides containing solution C. The sections are placed in a mixture of solution D: solution E: distilled water in a 1:1:2 ratio for 10 min, then washed twice with double‐distilled water at 4°C, each wash lasting 4 min. Next, the tissue is dehydrated in 50% ethanol for 4 min, followed by 75% and 95% ethanol, each for 40 s. Finally, the tissue is dehydrated in absolute ethanol for 4 min, repeating the cycle four times. The stained tissue is observed under a light microscope, and ImageJ software is used to calculate dendritic branching and the number of dendritic spines.

### 2.8 Hematoxylin–Eosin Staining (HE)

Stain the sections with hematoxylin for 5 min, followed by rinsing with double‐distilled water until there is no excess hematoxylin stain left. Differentiate the sections using a differentiation solution for 5 s. Afterward, immerse the sections in a bluing solution for bluing treatment. Dehydrate the sections sequentially in 85% and 95% ethanol for 5 min. Next, stain the sections with eosin for 5 min. After staining, dehydrate the sections in pure ethanol and mount them with neutral gum. Finally, observe the sections under a microscope and capture images for analysis.

### 2.9 Nissl Staining

Immerse the tissue sections in the staining solution for 5 min, followed by rinsing with water. Differentiate the sections slightly using 0.1% glacial acetic acid and the differentiation degree can be monitored and adjusted under a microscope. After rinsing with double‐distilled water, place the sections in an oven to dry. Next, immerse the sections in clean xylene for 10 min for clearing, and then mount them with neutral gum. Finally, observe the sections under a microscope and capture images for analysis.

### 2.10 Establishment of BV2‐HT‐22 Cell Co‐Culture Model and Drug Administration Method

Culture BV2 and HT22 cells in their respective culture media and allow them to grow for a period of time in a 37°C, 5% CO_2_ incubator. After this, use the embedded cell co‐culture method to co‐culture the two cell types, placing BV2 cells in the upper chamber and HT22 cells in the lower chamber. Treat BV2 cells with 200 μmol/L corticosterone for 24 h, then discard the supernatant and replace it with fresh culture medium for an additional 24 h incubation. This establishes a microglial and neuronal co‐culture model (Figure 9A).

### 2.11 Corticosterone(CORT)kits

The corticosterone level in the peripheral blood of the experimental mice was detected according to the instructions of the kit.

### 2.12 Surfacplasmon Resonance (SPR)

Rg3 was used as the analyte for 2× gradient dilution. A single‐cycle method was adopted. The above analyte flowed through the chip from low concentration to high concentration and bound to the target protein. The flow rate for each concentration was.

30 μL/min, the injection time was 120 s, the flow rate in the final dissociation section was 30 μL/min, and the detection duration was 600 s. The data were globally fitted to the 1:1 Langmuir binding model by using the Biacore Insight evaluation software (Cytiva, Marlborough, MA, USA) to obtain the binding and dissociation constants.

### 2.13 Statistical Analysis

Statistical analyses and graphical representations were conducted using OriginPro 2019 (OriginLab Corporation, USA) and GraphPad Prism 8 (GraphPad Software Inc., USA). For comparisons between two groups, either paired or unpaired Student's t‐tests were applied. For experiments involving multiple groups, one‐way or two‐way analysis of variance (ANOVA) was performed, following the Shapiro–Wilk test for normality. Post hoc analyses were conducted using either Bonferroni or Tukey's tests, as appropriate. Data are presented as the mean ± SEM, and statistical significance was defined as *p* < 0.05.

## 3 Results

### 3.1 The Complement Pathway and Synaptic Vesicle Cycle Undergo Changes in the CRS‐Induced Depression Mouse Model

**FIGURE 1:**
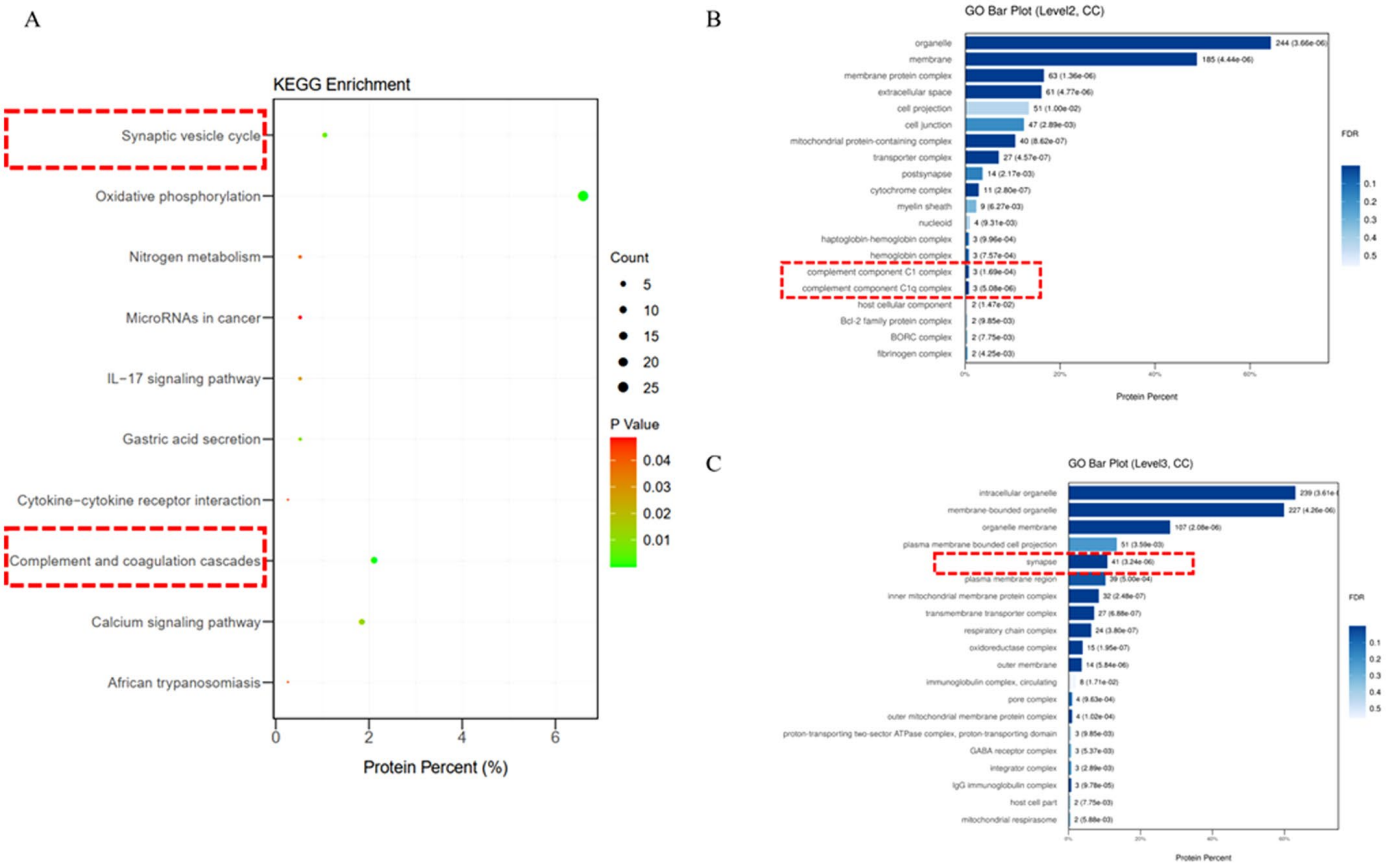
Changes in the complement pathway and synaptic vesicle cycling in the CRS‐induced depressive mouse model. (A) KEGG enrichment analysis was used to compare the differentially expressed proteins between the normal group and the depression group. (B, C) GO enrichment analysis of secreted proteins in the normal and depression groups.

After performing LC–MS/MS analysis on the hippocampal tissue of both the normal group and the depression model group of mice, significant differences were found between the two groups in several biological pathways, including the complement pathway and synaptic vesicle cycle. Through GO analysis and KEGG pathway enrichment analysis, we found that in the depression model group, the expression of complement‐related proteins, such as complement factor C1q, was significantly increased (Figure 1A–C).

### 3.2 The Active Components Related to Ginsenosides Interact With the C1q Molecule

**FIGURE 2:**
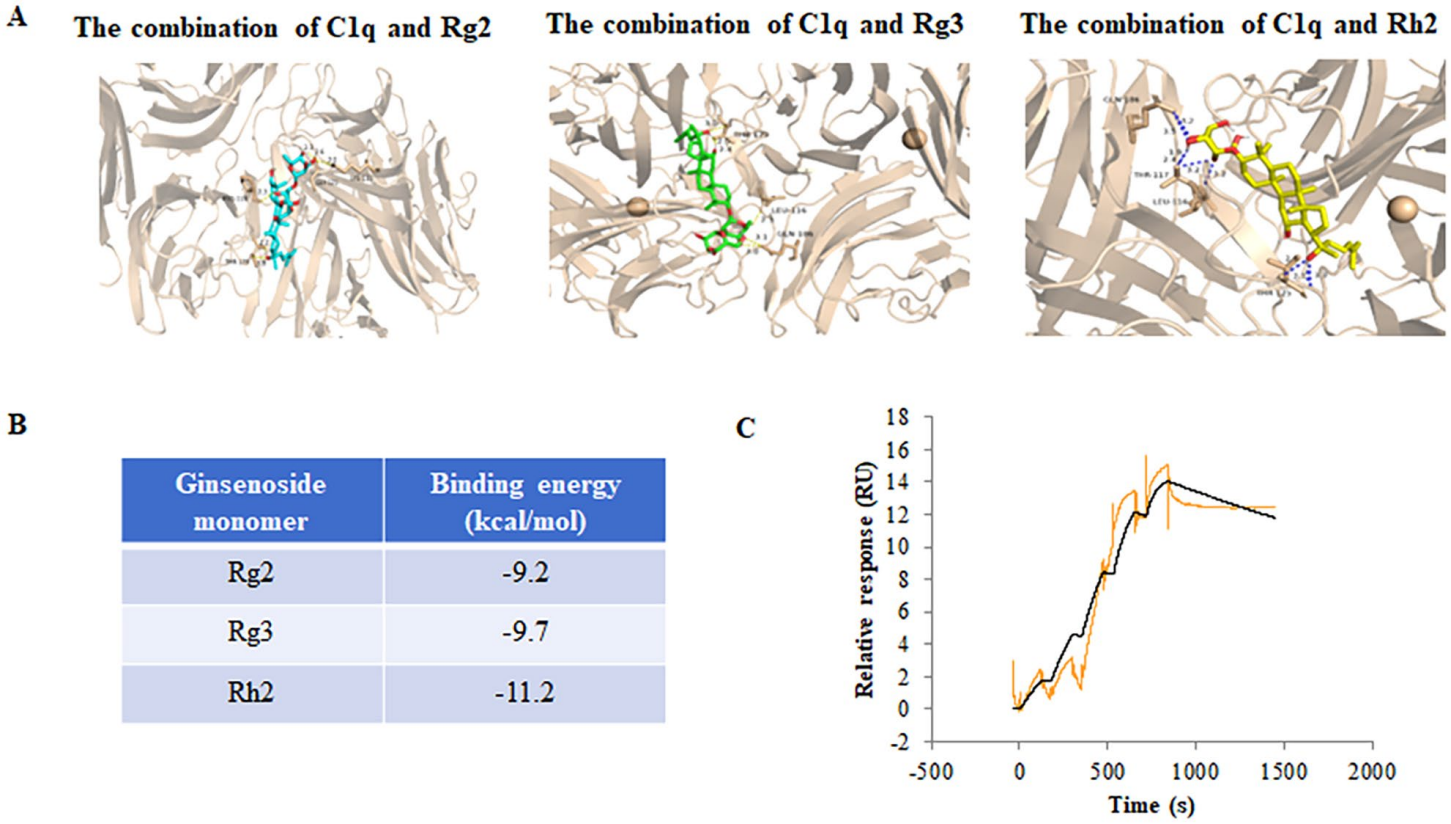
Interaction between active components related to ginsenosides and C1q receptor. (A) Experimental design for the binding of C1q with the three monomers of ginsenosides. (B) Binding energy values of the three monomers. (C) The affinity for the Rg3‐C1q interaction was determined using SPR.

Due to the presence of various types of saponins in ginsenosides, we conducted molecular docking experiments to verify the interaction between its main monomers and the C1q molecule. In the traditional Chinese medicine systems pharmacology database, we collected the major active components of ginsenosides and their related targets, screening for active molecules capable of binding to the C1q molecule. The active components of ginsenosides include Rg1, Rg2, Rg3, Rc, Rh2, and others. After docking with the C1q molecule, it was found that the binding energies of these molecules were all ≤ −5.0 kJ·mol^−1^, indicating stable binding with the target. We further selected the three active molecules with the lowest binding energies: Rg2, Rg3, and Rh2, with binding energies of −9.2, −9.7, and −11.2 kJ mol^−1^, respectively (Figure 2A,B). SPR results indicated that C1q interacted with Rg3, and the affinity between the two was 1.65 μM (Figure 2C).

### 3.3 Comparison of the Antidepressant Effects of Ginsenoside Rg3 and Rh2

**FIGURE 3:**
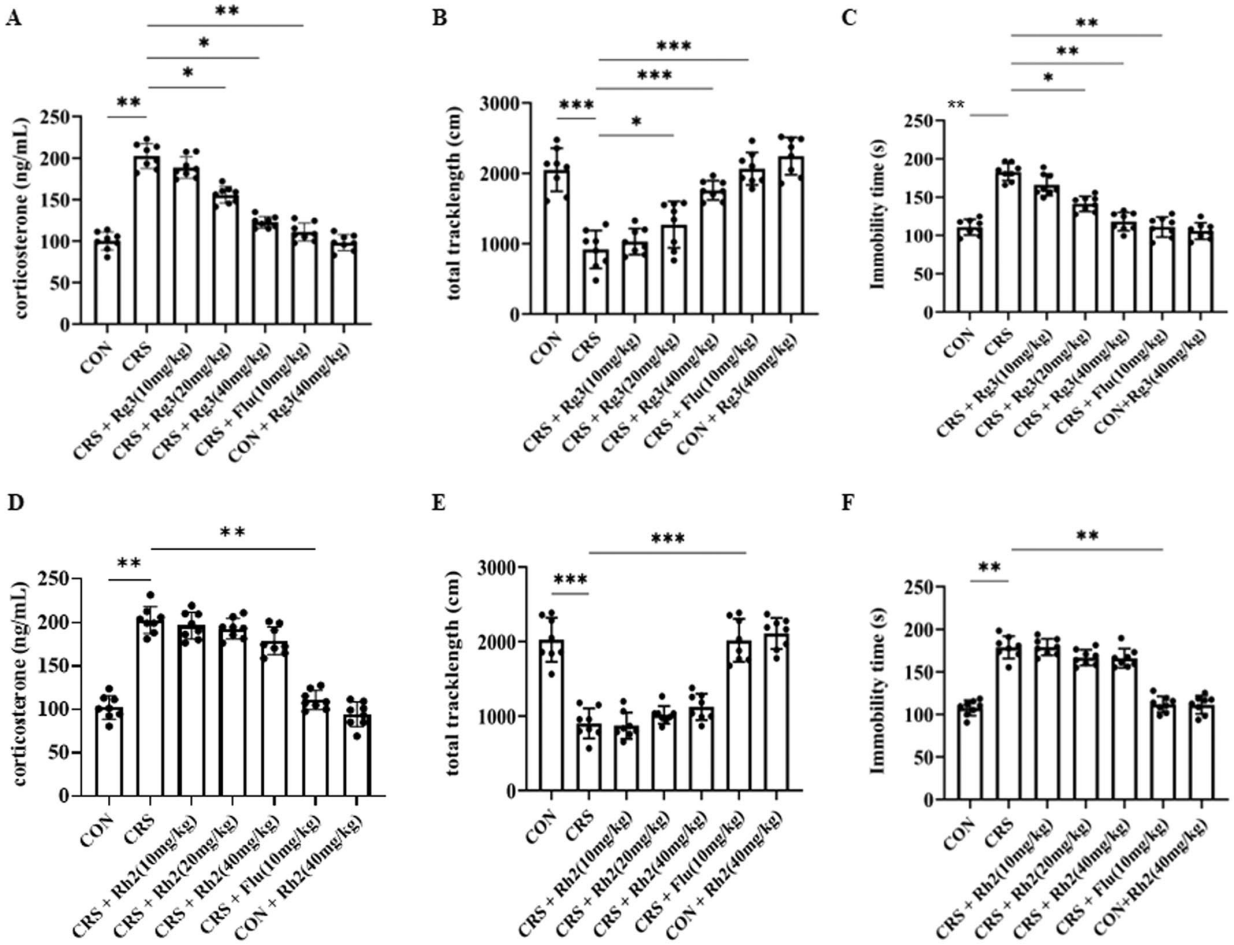
Comparison of the antidepressant effects of ginsenoside Rg3 and Rh2. (A) ELISA analysis of the effect of ginsenoside Rg3 on the serum CORT levels. (B) Effect of ginsenoside Rg3 on the open field test. (C) Effect of ginsenoside Rg3 on immobility time in the forced swimming test. (D) ELISA analysis of the effect of ginsenoside Rh2 on the serum CORT levels. (E) Effect of ginsenoside Rh2 on the open field test. (F) Effect of ginsenoside Rh2 on immobility time in the forced swimming test. All data are presented as the mean ± SEM. *p < 0.05, **p < 0.01, ***p < 0.001 versus the indicated group.

Based on the molecular docking results, we selected the stronger monomers, ginsenosides Rg3 and Rh2. To compare their effects on CORT levels, we used an ELISA kit to measure the corticosterone content in the serum of mice from each group. The results showed that, compared to the CON group, the CRS group had a significant increase in corticosterone levels. Compared to the CRS group, after treatment with ginsenoside Rg3, the corticosterone levels decreased in both the medium and high‐dose groups, and the fluoxetine group also showed a significant decrease (Figure 3A). However, treatment with ginsenoside Rh2 showed only a slight trend toward reduced corticosterone levels (Figure 3D). Compared with the CRS group, the total movement distance of mice in the ginsenoside Rg3 group increased, and the forced swimming immobility time was significantly reduced (Figure 3B,C). Compared with the CRS group, there were no significant changes in the total movement distance and forced swimming immobility time of mice in the ginsenoside Rh2 group (Figure 3E,F). Therefore, we selected ginsenoside Rg3 for further research on the treatment of depression in mice.

### 3.4 Effect of Ginsenoside Rg3 on Behavioral Changes in CRS‐Induced Mice

**FIGURE 4:**
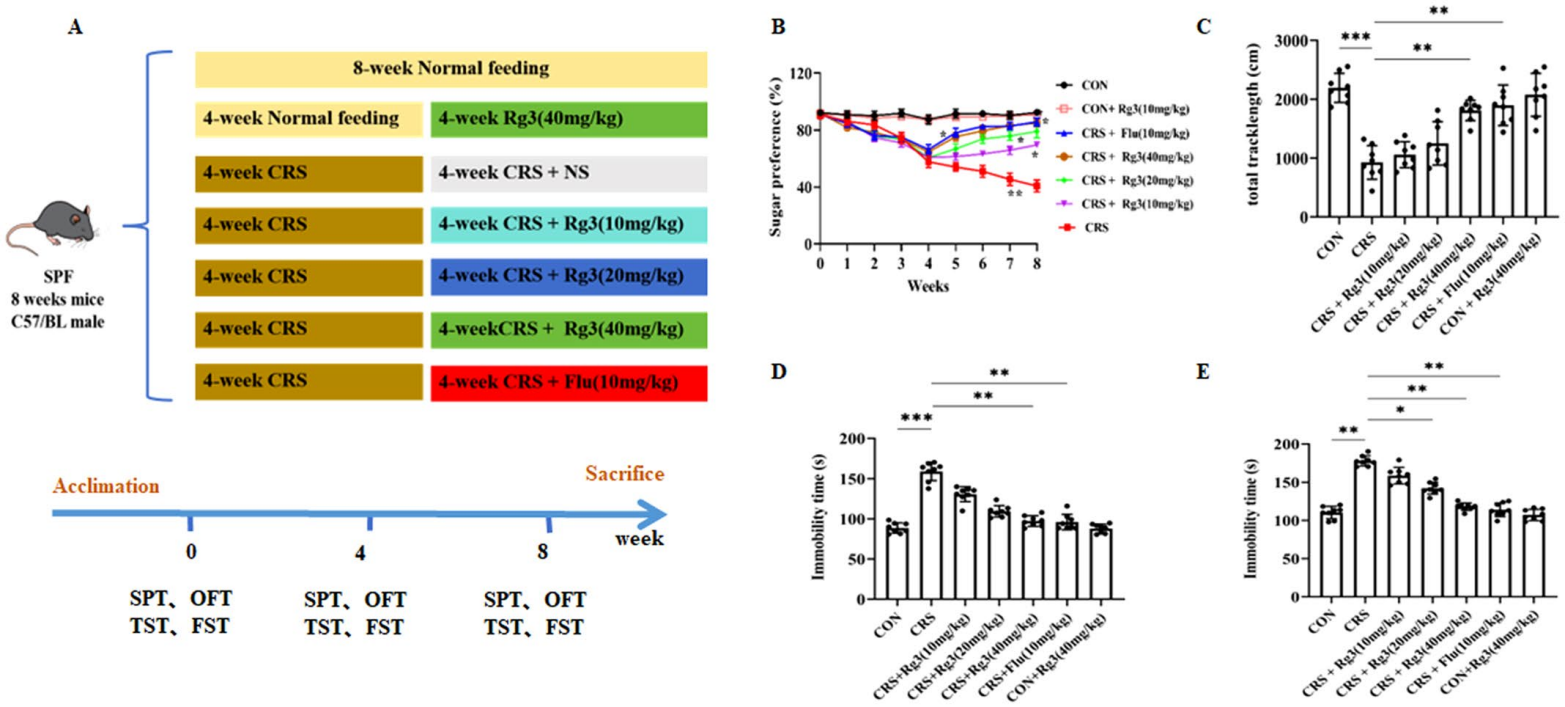
Effect of Ginsenoside Rg3 on behavioral changes in CRS‐induced mice. (A) Diagram of the treatment protocol. (B) Effect of ginsenoside Rg3 on sucrose preference rate. (C) Effect of ginsenoside Rg3 on the open field test. (D) Effect of ginsenoside Rg3 on immobility time in the forced swimming test. (E) Effect of ginsenoside Rg3 on immobility time in the tail suspension test. All data are presented as the mean ± SEM. *p < 0.05, **p < 0.01, ***p < 0.001 versus the indicated group.

The behavioral research was carried out in accordance with the experimental scheme in Figure 4A. The results showed that, compared to the CON group, the sucrose preference rate in the CRS group was significantly reduced. After treatment with ginsenoside Rg3, the sucrose preference rate was restored in the different concentrations of ginsenoside Rg3 groups compared to the CRS model group, and the fluoxetine group also showed a significant increase (Figure 4B). The open field test results showed that, compared to the CON group, the total distance moved by the CRS group mice in the open field was significantly reduced. After drug intervention, compared to the CRS group, the total distance moved by the mice in the high‐dose ginsenoside Rg3 group was increased, and the fluoxetine group showed a significant increase in total movement distance (Figure 4C). The forced swimming test results indicated that, compared to the CON group, the immobility time of the CRS group mice was significantly increased. Compared to the CRS group, the immobility time in the high‐dose ginsenoside Rg3 group and the fluoxetine group was significantly reduced (Figure 4D). The tail suspension test showed that, compared to the normal control group, the cumulative immobility time of the CRS model group mice was significantly increased. Compared to the CRS model group, the cumulative immobility time in the medium and high‐dose ginsenoside Rg3 groups was significantly reduced, and the fluoxetine group also showed a significant decrease in cumulative immobility time (Figure 4E). In summary, the high‐dose ginsenoside Rg3 group demonstrated the best antidepressant effect, significantly improving the depressive‐like behavior induced by CRS in mice.

### 3.5 Effect of Ginsenoside Rg3 on the Pathological Morphology of Hippocampal Neurons in CRS‐Induced Mice

**FIGURE 5:**
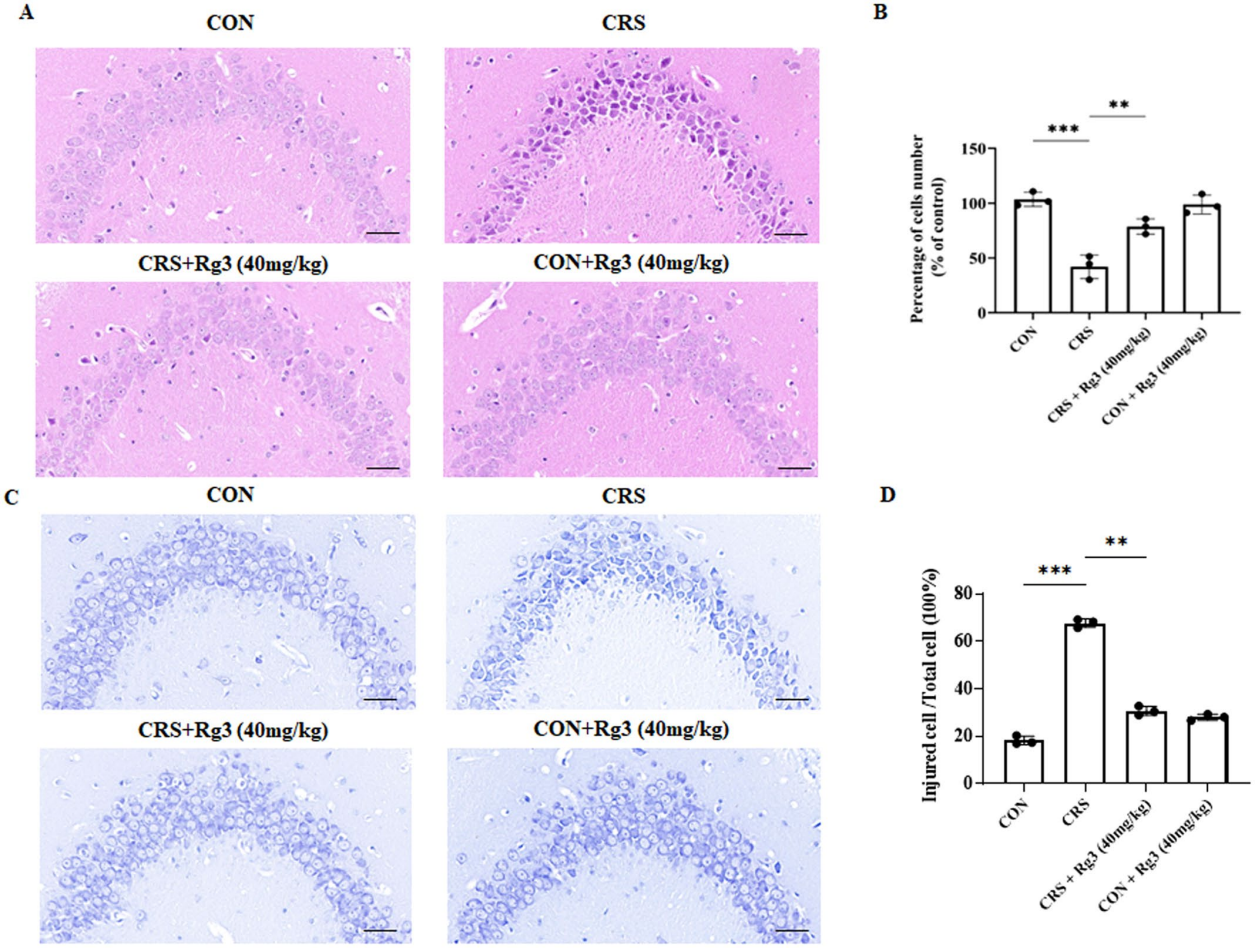
Effect of Ginsenoside Rg3 on the pathological morphology of hippocampal neurons in CRS‐induced mice. (A) HE staining results of mouse hippocampus, scale bar: 50 μm. (B) Quantification of HE staining results. (C) Nissl staining results of mouse hippocampus, scale bar: 100 μm. (D) Quantification of Nissl staining results. All data are presented as the mean ± SEM. **p < 0.01, ***p < 0.001 versus the indicated group.

Neuron damage is considered one of the main pathological changes in depression, and patients with depression often exhibit changes in hippocampal neurons. Compared to the CON group, the hippocampal CA3 region neurons in the CRS group exhibited nuclear pyknosis, cytoplasmic condensation, disorganized cell arrangement, and some cells were triangular or spindle‐shaped. Compared to the CRS group, the CRS + Rg3 (40 mg/kg) group showed more closely packed and organized hippocampal neurons, with reduced damage (Figure 5A,B). Nissl body staining results showed that, compared to the CON group, the CRS group had fewer Nissl body granules in the hippocampal CA3 region, altered cell morphology, and lighter cytoplasmic staining. Compared to the CRS group, the CRS + Rg3 (40 mg/kg) group had an increase in Nissl body granules, and the neuronal morphology appeared more normal (Figure 5C,D). Additionally, no changes in neuronal morphology were observed in the CON+Rg3 (40 mg/kg) group. These results suggest that ginsenoside Rg3 can alleviate hippocampal neuronal damage induced by CRS.

### 3.6 The Effect of Ginsenoside Rg3 on Microglial Cells and Complement Factor C1q Levels

**FIGURE 6:**
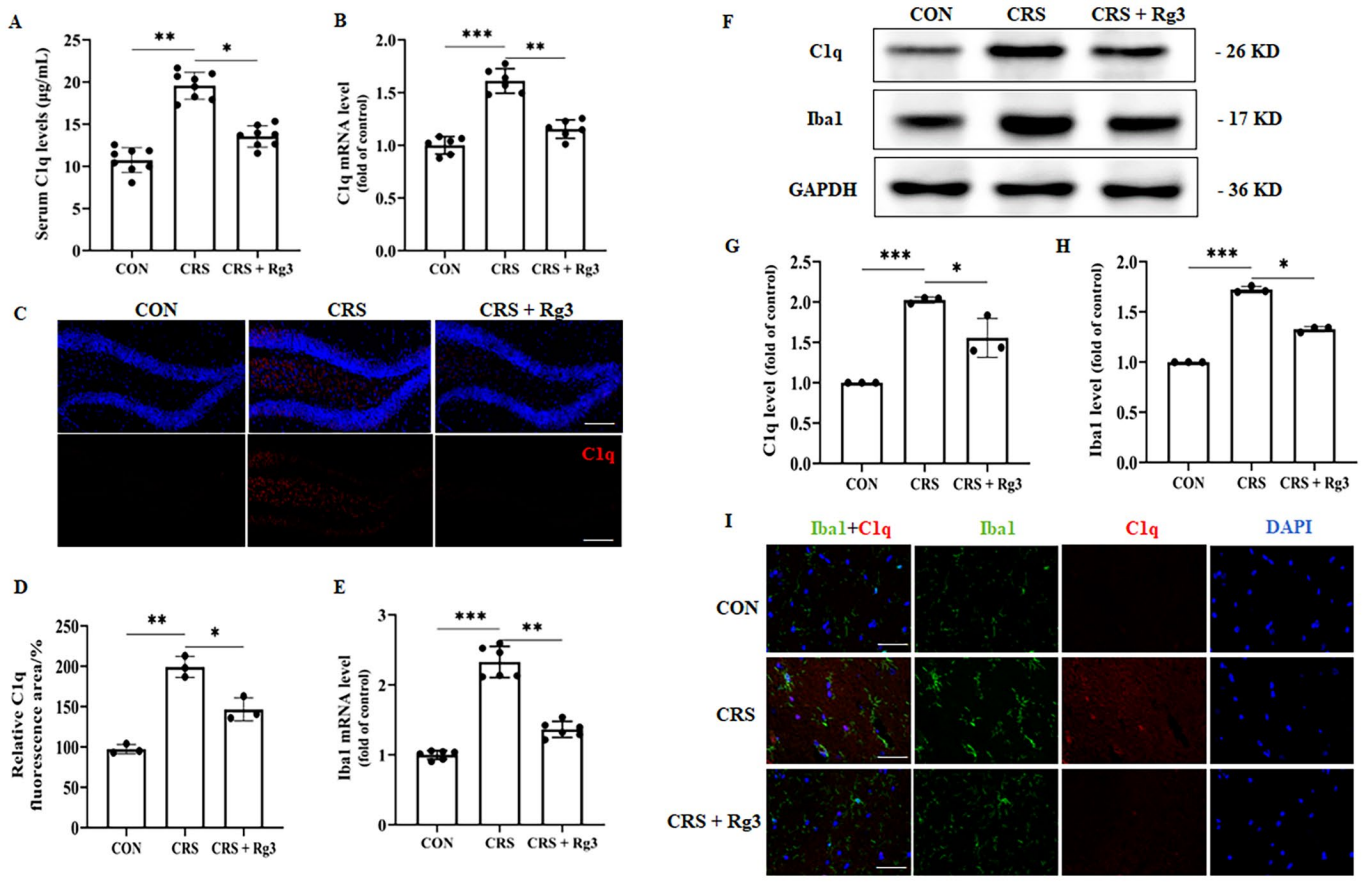
The effect of ginsenoside Rg3 on microglial cells and complement factor C1q levels. (A) Detection of complement factor C1q content in mouse peripheral blood using an ELISA kit. (B) RT‐qPCR analysis of C1q mRNA expression in hippocampal tissue. (C) Immunofluorescence results in the mouse hippocampal region, scale bar: 50 μm. (D) Quantification of immunofluorescence. (E) RT‐qPCR analysis of Iba1 mRNA expression in hippocampal tissue. (F) Western blot analysis of C1q and Iba1 protein expression. (G‐H) Protein Expression Quantification Chart. (I) Immunofluorescence double staining of Iba1 and C1q, scale bar: 100 μm. All data are presented as the mean ± SEM. *p < 0.05, **p < 0.01, ***p < 0.001 versus the indicated group.

Previous experiments have demonstrated that ginsenoside Rg3 has certain antidepressant effects. To investigate the impact of ginsenoside Rg3 on hippocampal microglial cells and complement factor C1q levels in the CRS‐induced depression mouse model. The results showed that, compared to the CON group, the C1q level in the peripheral blood of the CRS group was significantly increased. After treatment with ginsenoside Rg3, the expression of C1q was suppressed in the CRS group (Figure 6A). Compared to the CON group, the C1q mRNA level in the hippocampal tissue of the CRS group was significantly elevated. After ginsenoside Rg3 treatment, the elevated C1q mRNA level was significantly suppressed (Figure 6B). Compared to the CON group, C1q levels were significantly increased in the CRS group. After ginsenoside Rg3 treatment, the C1q levels were reduced compared to the CRS group (Figure 6C,D,F,G). These results confirm that ginsenoside Rg3 significantly reduces the level of complement factor C1q. Compared to the CON group, the Iba1 mRNA level in the CRS group was significantly elevated. After ginsenoside Rg3 treatment, the Iba1 mRNA level was significantly reduced in the CRS group (Figure 6E). Similarly, WB results showed that, compared to the CON group, the Iba1 level was significantly elevated in the CRS group. After ginsenoside Rg3 treatment, the Iba1 level was decreased compared to the CRS group (Figure 6F,H). Additionally, the number of C1q‐positive microglial phagocytic puncta in the hippocampal region of the CRS model group was increased, while after ginsenoside Rg3 treatment, the number was significantly reduced (Figure 6I). These data suggest that ginsenoside Rg3 can lower the levels of C1q and inhibit the activation of microglial cells.

### 3.7 Effect of Ginsenoside Rg3 on Dendritic Spines of Hippocampal Neurons in Mice

**FIGURE 7:**
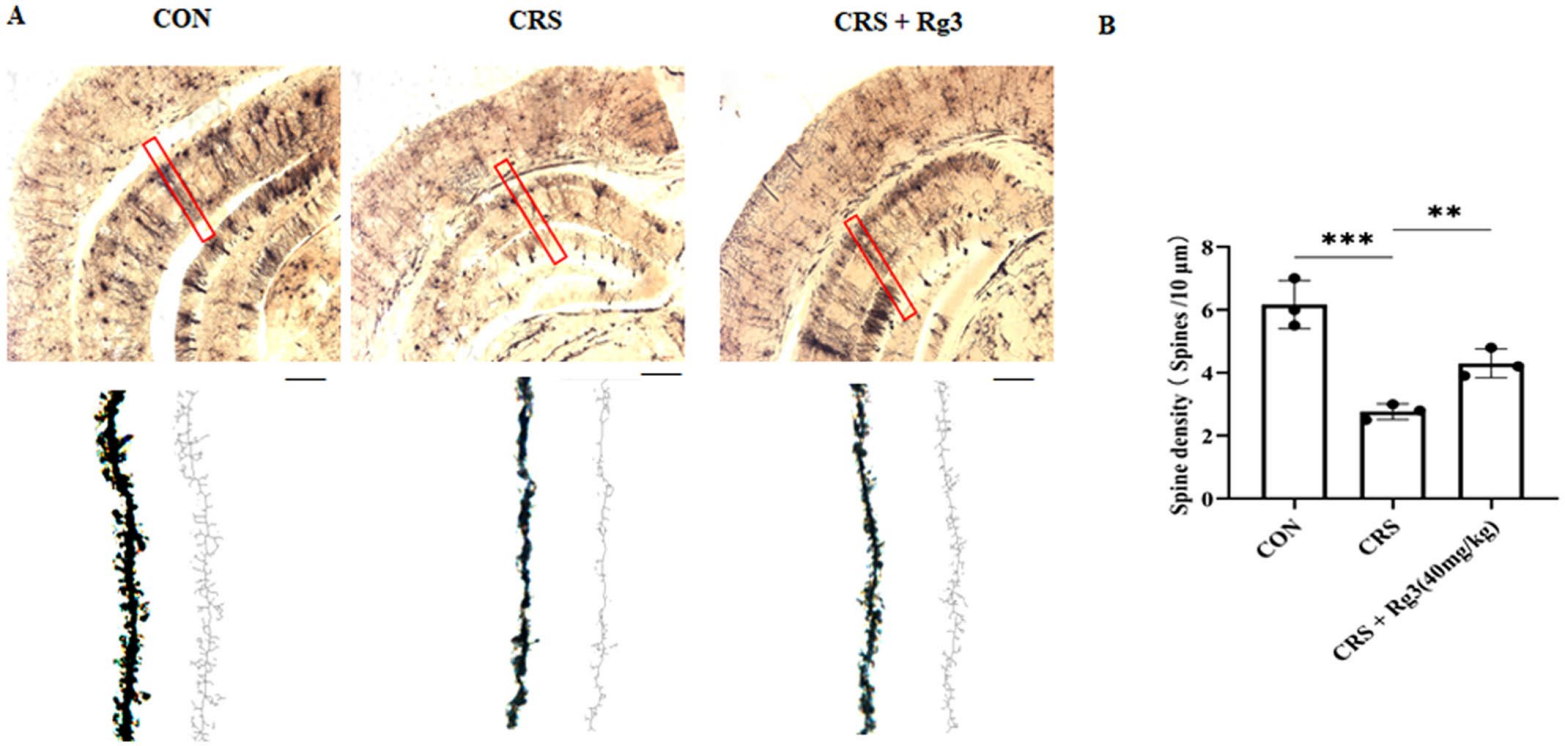
Effect of Ginsenoside Rg3 on dendritic spines of hippocampal neurons in mice. (A) Golgi staining results of mouse hippocampus, scale bar: 100 μm. (B) Quantification of Golgi staining results. All data are presented as the mean ± SEM. **p < 0.01, ***p < 0.001 versus the indicated group.

In clinical settings, most patients with depression exhibit dendritic damage. Golgi staining can be used to detect the density of dendritic spines in the hippocampal region of mice. The results of the Golgi staining analysis showed that, compared to the CON group, the CRS group mice exhibited a significant reduction in the number of dendritic spines in the hippocampal region. However, in the ginsenoside Rg3 treatment groups, the number of dendritic spines in the hippocampal region was significantly increased compared to the CRS group (Figure 7A,B). These experimental results indicate that ginsenoside Rg3 treatment improves the dendritic damage in neurons induced by CRS in mice.

### 3.8 Effect of Ginsenoside Rg3 on Microglial Phagocytic Function

**FIGURE 8:**
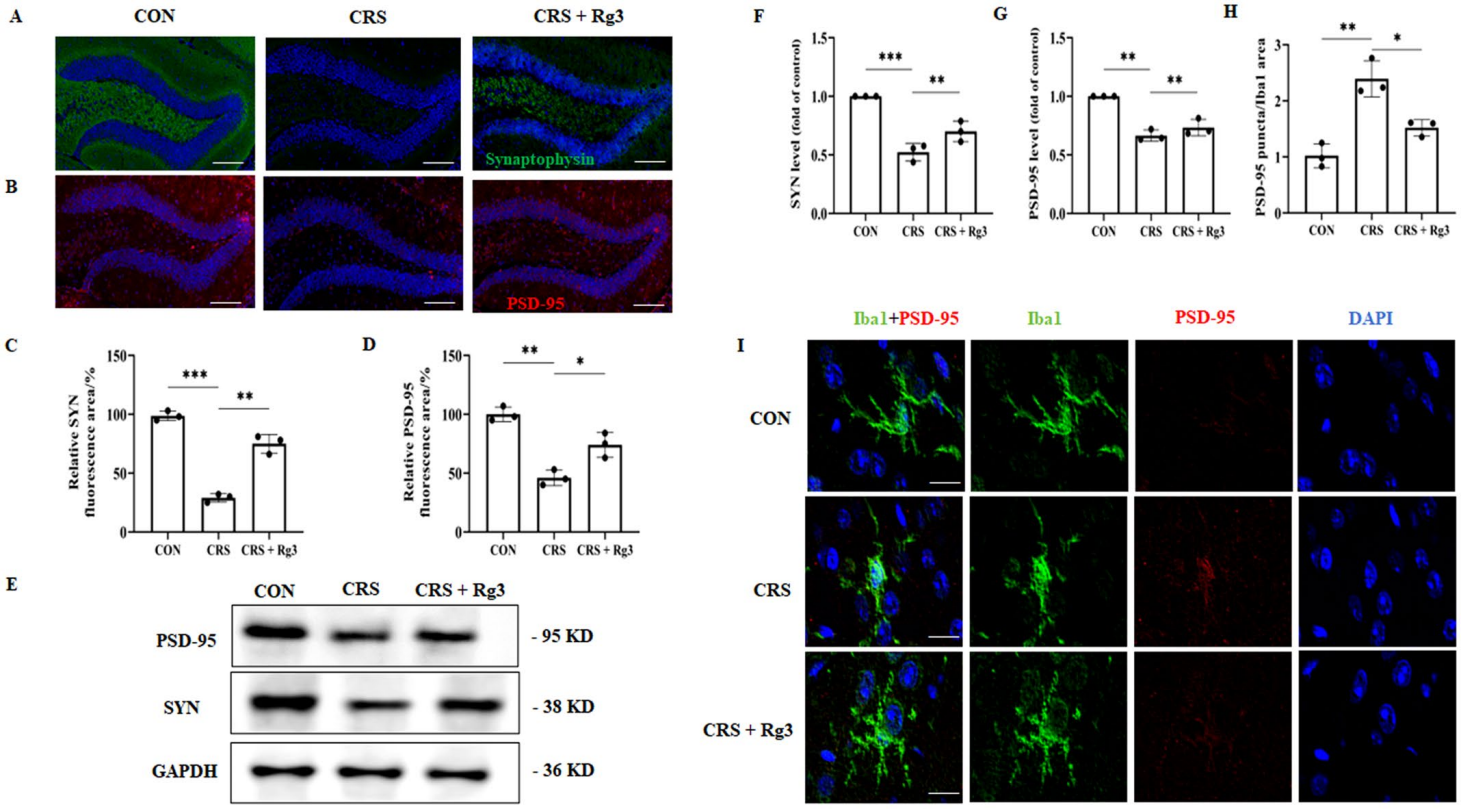
Effect of Ginsenoside Rg3 on microglial phagocytic function. (A, B) Immunofluorescence staining results, scale bar: 50 μm. (C, D) Immunofluorescence quantification chart. (E) WB detection of PSD‐95 and SYN protein expression. (F, G) Protein expression quantification chart. (H) Quantitative analysis of PSD‐95 phagocytic puncta in microglial cells. (I) Representative immunofluorescence staining of PSD‐95 co‐labeled with Iba1 in the hippocampal region, scale bar: 200 μm. All data are presented as the mean ± SEM. *p < 0.05, **p < 0.01, ***p < 0.001 versus the indicated group.

To further observe the effect of ginsenoside Rg3 on synapse‐related protein expression, hippocampal tissue was collected for immunofluorescence experiments. The results showed that, compared to the CON group, the expression of PSD‐95 and SYN in the CRS group was significantly decreased. However, in the ginsenoside Rg3 treatment group, the expression of PSD‐95 and SYN was significantly increased compared to the CRS group (Figure 8A–D). Western blot results indicated that, compared to the CON group, the expression of PSD‐95 and SYN was significantly reduced in the CRS group. In contrast, in the ginsenoside Rg3 treatment group, the expression of PSD‐95 and SYN was notably higher than that in the CRS group (Figure 8E–G). These results confirm that synaptic loss occurred in the hippocampal region of the CRS group, and ginsenoside Rg3 can improve synaptic defects. In addition, the results revealed that, compared to the CON group, the number of PSD‐95 phagocytic puncta within microglial cells in the CRS group was significantly increased. In contrast, in the ginsenoside Rg3 treatment group, the number of PSD‐95 phagocytic puncta within microglial cells was significantly reduced compared to the CRS group (Figure 8H,I). These results suggest that ginsenoside Rg3 can effectively inhibit the abnormal phagocytosis of synapses by microglial cells and restore the levels of PSD‐95 and SYN in the hippocampal region.

### 3.9 Effect of Ginsenoside Rg3 on the Level of Complement Factor C1q in BV2 Cells

**FIGURE 9:**
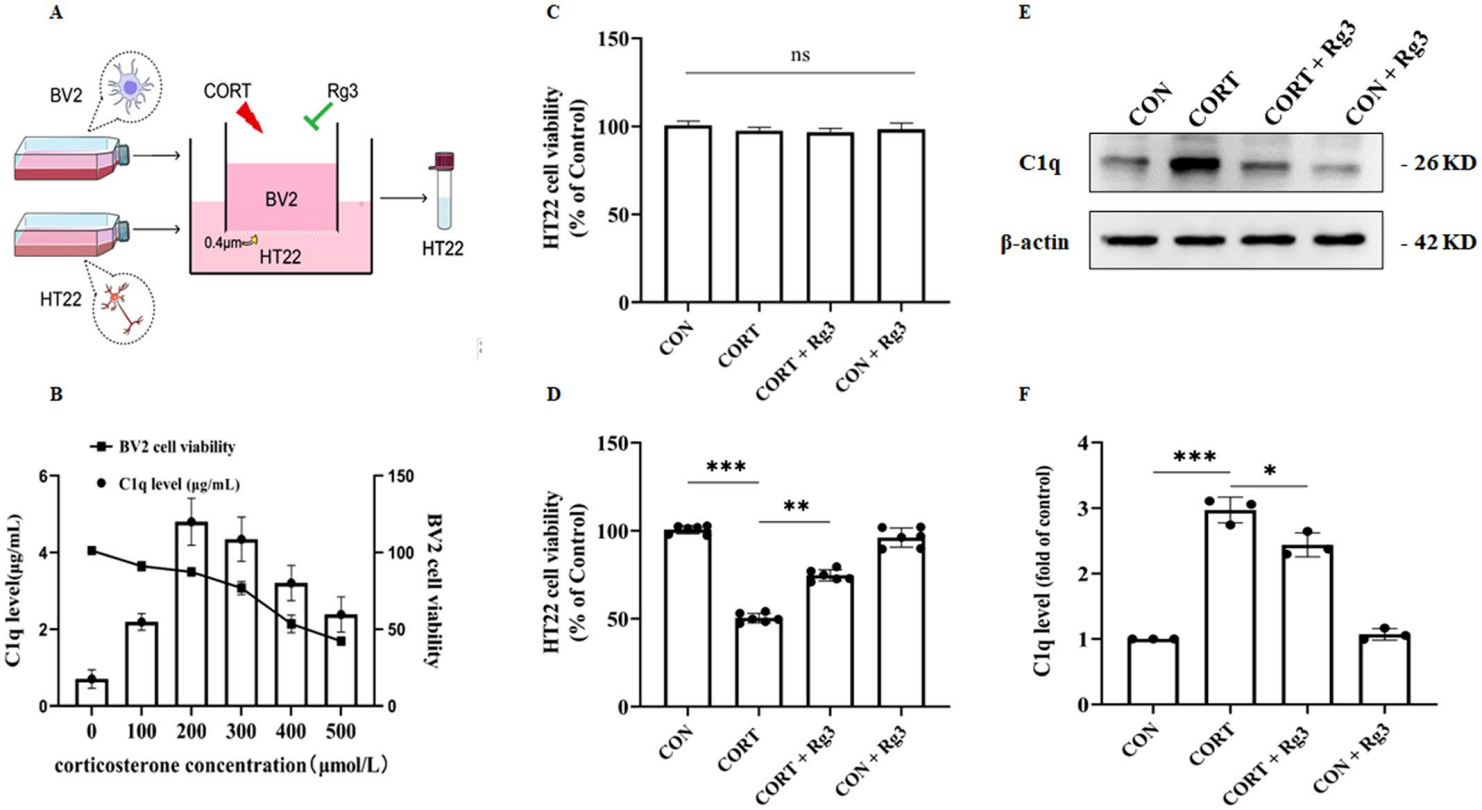
Effect of Ginsenoside Rg3 on the level of complement factor C1q in BV2 cells. (A) Schematic of the co‐culture model of microglial cells and neuronal cells. (B) The effects of different concentrations of corticosterone on BV2 cell viability and complement factor levels. (C‐D) CCK‐8 assay results showing HT22 cell viability. (E) Expression of C1q protein. (F) Quantification of protein expression results. All data are presented as the mean ± SEM. *p < 0.05, **p < 0.01, ***p < 0.001 versus the indicated group.

We cultured BV2 and HT22 using a co‐culture system with Transwell (Figure 9A). The results showed that when the concentration of CORT is 200 μmol/L, the BV2 cells were minimally damaged and secreted the highest levels of C1q (Figure 9B). Subsequent experiments used this concentration of CORT to directly stimulate the HT22 cells. The results indicated that there was no significant neuronal death, and there were no statistical differences across the four groups. When activated BV2 cell supernatant was used to stimulate HT22 cells, the survival rate of HT22 cells in the CON and Rg3 groups did not show significant changes. However, compared to the CON group, the survival rate of HT22 cells in the CORT group was significantly decreased. In contrast, in the CORT+Rg3 group, the survival rate of HT22 cells significantly increased compared to the CORT group, and the difference was statistically significant (Figure 9C,D). However, in the CORT+Rg3 group, C1q expression was significantly reduced compared to the CORT group (Figure 9E,F). These results suggest that ginsenoside Rg3 may exert its antidepressant effects by modulating C1q expression, possibly by inhibiting the activation of microglial cells and thereby protecting neuronal cells from CORT‐induced damage.

### 3.10 Effect of Ginsenoside Rg3 on Synapses of HT22 Neurons

**FIGURE 10:**
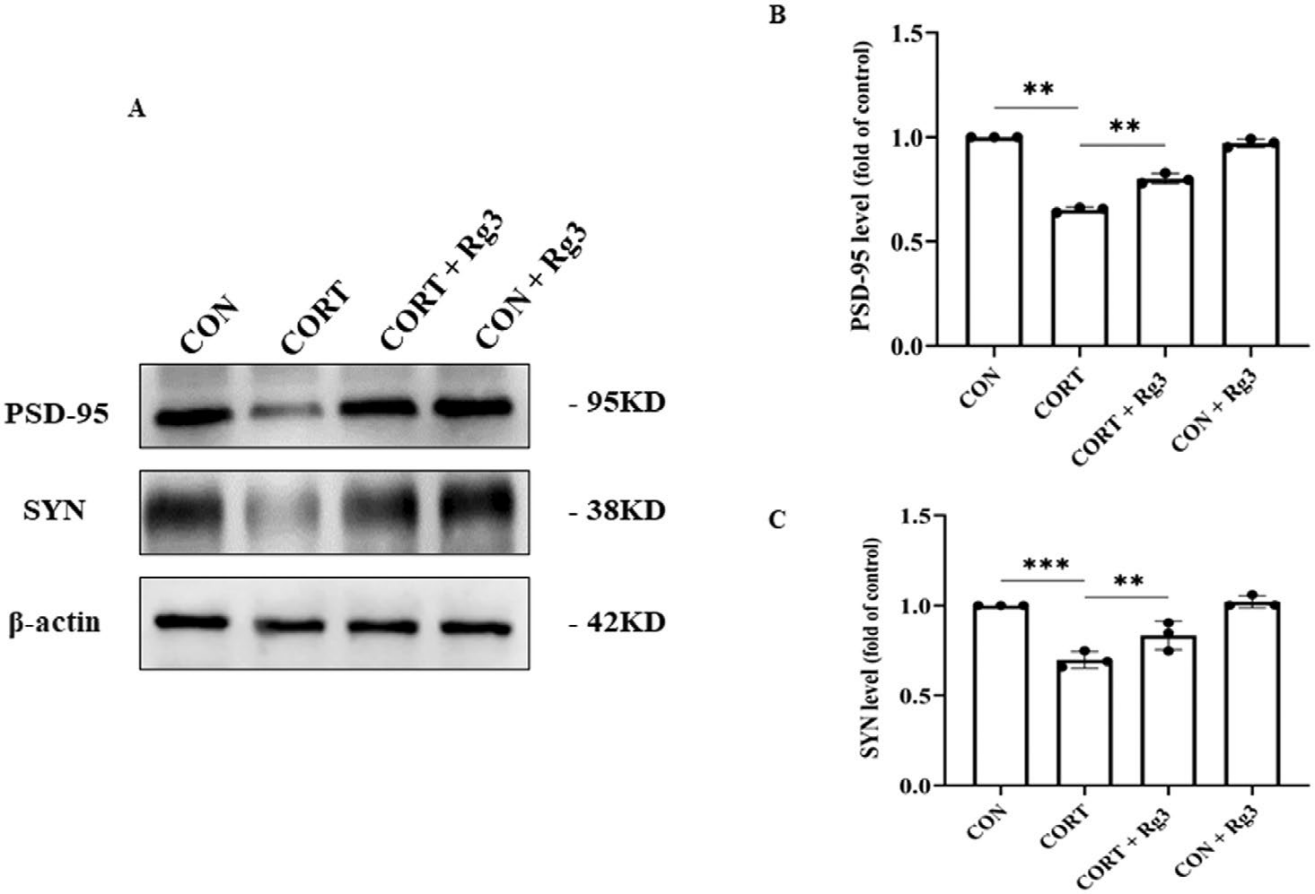
Effect of Ginsenoside Rg3 on synapses of HT22 neurons. (A) Expression of synaptic‐related proteins. (B‐C) Quantification of protein results. All data are presented as the mean ± SEM. **p < 0.01, ***p < 0.001 versus the indicated group.

Compared to the CON group, the expression of PSD‐95 in the CORT group was significantly reduced. However, in the CORT+Rg3 group, the expression of PSD‐95 was significantly increased compared to the CORT group. Similarly, compared to the CON group, the expression of SYN in the CORT group was significantly decreased. In contrast, in the CORT+Rg3 group, the expression of SYN was significantly higher compared to the CORT group (Figure 10A–C). These findings suggest that ginsenoside Rg3 can reduce the loss of synapses in HT22 cells, likely by improving the expression of key synaptic proteins such as PSD‐95 and SYN.

### 3.11 Effect of Ginsenoside Rg3 on BV2 Cell Supernatant‐Induced Apoptosis in HT22 Neurons

**FIGURE 11:**
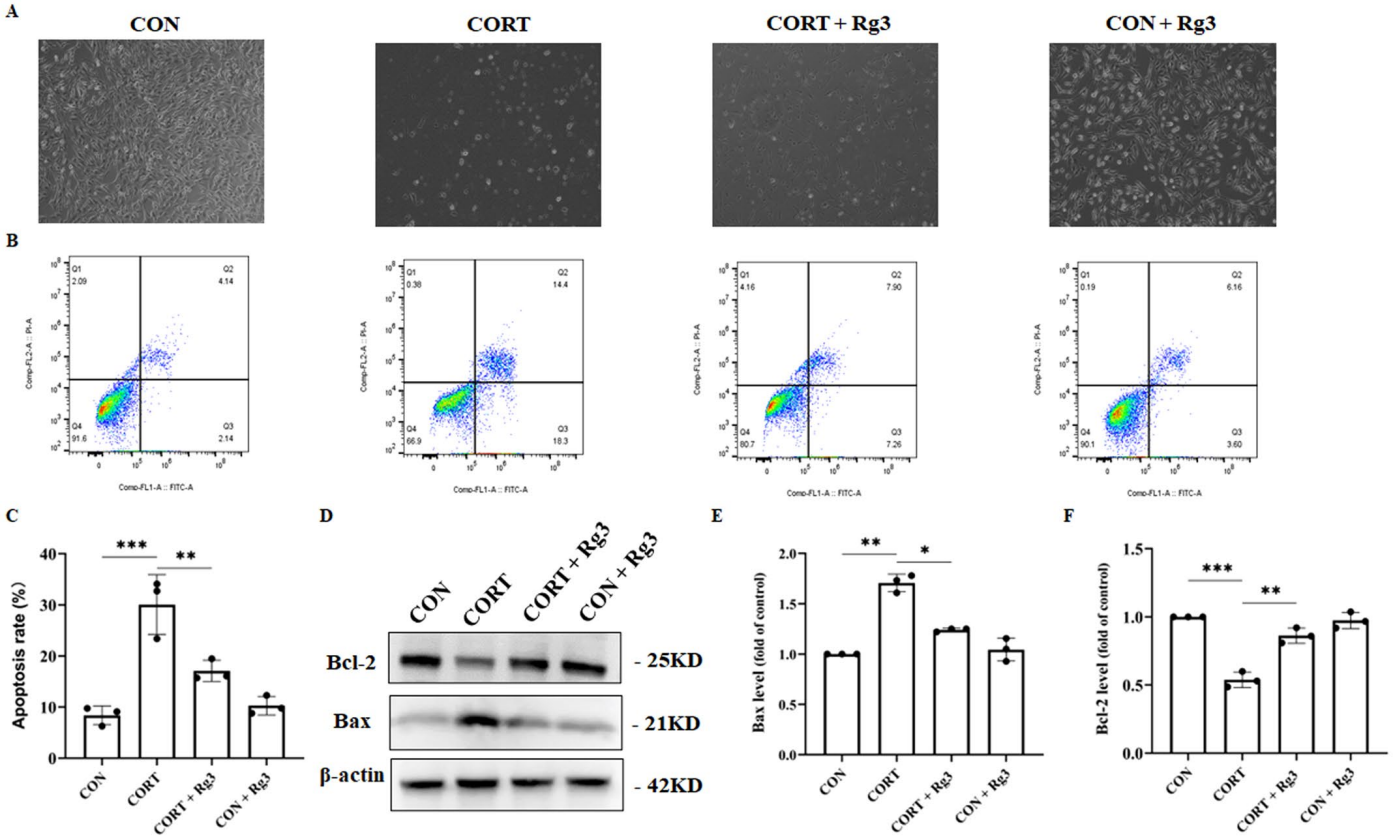
Effect of ginsenoside Rg3 on BV2 cell supernatant‐induced apoptosis in HT22 neurons. (A) Morphological observation of HT22 cells under a microscope. (B) Flow cytometry detection of apoptosis rate in HT22 cells. (C) Quantification of HT22 apoptosis rate. (D) Expression of apoptosis‐related proteins. (E, F) Quantification of protein expression. All data are presented as the mean ± SEM. *p < 0.05, **p < 0.01, ***p < 0.001 versus the indicated group.

Compared to the CON group, the CORT group showed a significant reduction in the number of cells, with most of the cell processes disappearing and the cells becoming round (Figure 11A). In the CORT+Rg3 group, the number of cells showed some increase compared to the CORT group. Flow cytometry results revealed that the apoptosis rate of HT22 cells in the CORT group was significantly higher than in the CON group. However, in the CORT+Rg3 group, the apoptosis rate of HT22 cells was significantly lower compared to the CORT group (Figure 11B,C). Western blot analysis showed that, compared to the CON group, the expression of Bcl‐2 was significantly reduced, while the expression of Bax was significantly increased in the CORT group. In contrast, in the CORT+Rg3 group, the expression of Bcl‐2 was significantly higher, and the expression of Bax was significantly lower compared to the CORT group. The expression of Bcl‐2 and Bax in the CON+Rg3 group did not show significant differences compared to the CON group (Figure 11D–F).

**FIGURE 12:**
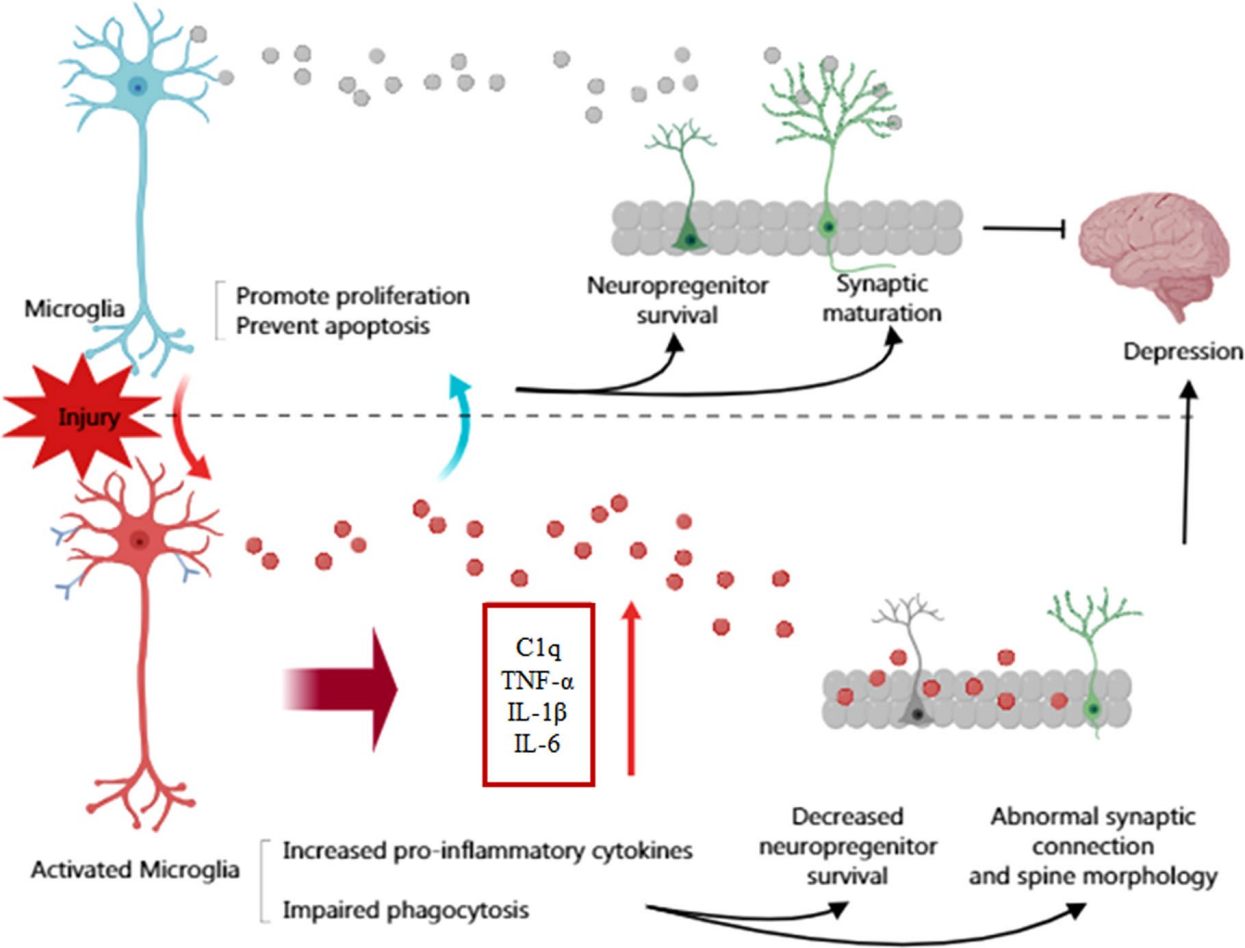
Ginsenoside Rg3 may reduce complement factor secretion and inhibit neuronal apoptosis by regulating microglial activation, thereby exerting a protective effect on neurons.

In conclusion, the chronic restraint model can induce depressive behaviors in mice and hippocampal microglia secrete complement factor C1q. Complement factors mediate microglial activation and their phagocytic function in a chronic inflammatory environment. Ginsenoside Rg3 may reduce complement factor secretion and inhibit neuronal apoptosis by regulating microglial activation, thereby exerting a protective effect on neurons (Figure 12).

## 4 Discussion

Depression is a psychological disorder primarily characterized by loss of interest and low mood, which is widespread globally and has become a serious public health issue [19]. Significant increases in the complement factor C1q have been observed in both depression patients and animal experiments [20, 21]. The complement system is a critical component of the immune system, responsible for clearing cellular debris, infection sources, and regulating immune responses. C1q is the first component of the complement system, which usually activates the complement cascade upon binding with antibodies. It plays an important role in immune responses, clearing apoptotic cells, and regulating local inflammatory responses [22]. In our study, we first found significant abnormalities in the complement pathway and synaptic vesicle cycling in the brain regions of CRS model mice. The depression model induced by CRS is usually accompanied by changes in neuroinflammation and immune responses, with the complement system playing a crucial role in this process. Studies show that C1q, as a key factor in the complement system, regulates neuroimmune responses in the central nervous system, thereby affecting synaptic function and the stability of neural networks [23]. Therefore, the abnormal increase in C1q could be an important mechanism in the occurrence and development of depression. Additionally, microglial cells are considered the main source of C1q. When the body is in a depressed state, microglial cells become activated and secrete various related factors, including complement factor C1q. By releasing C1q, microglial cells may affect synaptic function and morphology, even triggering synaptic pruning [24, 25, 26]. Although synaptic pruning is a normal phenomenon during neural development, excessive or abnormal pruning is associated with various neuropsychiatric diseases, including depression [27]. Our research clarified that the C1q in the hippocampal brain region of the bound model mice was upregulated, microglia were abnormally activated, and neuronal synapses were lost, thereby causing depressive‐like behaviors in the mice, which is consistent with the previous research results.

Ginsenoside Rg3, an active ingredient extracted from ginseng, belongs to the ginsenoside class of compounds [28, 29]. As one of the main active ingredients in ginseng, ginsenosides exhibit various biological activities, including antioxidant, anti‐inflammatory, and immune‐regulatory properties [30]. In recent years, research has increasingly focused on exploring the potential effects of Rg3 in neurodegenerative diseases, especially depression. A study by Yang J and colleagues explored the effects of Ginsenoside Rg3 in a mouse depression model and found that Rg3 significantly alleviated depressive‐like behavior by modulating the 5‐HT and DA systems, providing a neurobiological basis for its antidepressant effects and supporting its potential as a therapeutic drug [31]. Another study by Zhang M reviewed clinical studies on ginseng and its main active components (such as Rg1, Rb1, Rg3) in the treatment of depression, showing that, despite the small sample size, these components have some efficacy in alleviating depressive symptoms [32]. Wang X and colleagues studied the effects of Rg3 in an LPS‐induced depression rat model and found that Rg3 significantly improved depressive symptoms by inhibiting inflammation and regulating immune function [33]. Furthermore, Zhang M's research suggests that Rg3 improves chronic stress‐induced depressive‐like behaviors by increasing BDNF expression and promoting neural plasticity, providing new insights into its antidepressant mechanism [34]. Additionally, research by Yuan T and colleagues indicated that prolonged exposure to elevated corticosterone in the amygdala central nucleus leads to visceral hypersensitivity and morphological changes in microglial cells, with increased expression of C1q and CR3, thereby promoting enhanced synaptic phagocytosis. Using a CR3 antagonist could reverse these synaptic changes and alleviate disease progression [35]. Previous studies have shown that Rg3 can pass through the blood–brain barrier after oral administration, and its half‐life is 4–6 h [36]. Most of these studies focus on its role in regulating inflammatory factors and neurotransmitters, with less discussion on its regulation of the complement system. In this study, no weight loss or organ pathological damage was observed in the high‐dose group (40 mg/kg), which was consistent with the safety reported in the literature [37]. We first confirmed that ginsenoside Rg3 improves depressive‐like behaviors in mice by inhibiting the upregulation of C1q, reducing abnormal activation of microglia, and alleviating the loss of neuronal synapses.

Our research clarifies that the upregulation of C1q is a key target for the neuroprotective effect of Rg3. Therefore, we propose that Rg3 improves depressive‐like behaviors in mice by inhibiting the upregulation of C1q, reducing abnormal activation of microglia, and alleviating the loss of neuronal synapses. Our research also has some limitations. It has not been directly proved that C1q is the only and necessary downstream effector molecule for the action of Rg3. It is necessary to knockout or overexpress C1q in microglia, and then observe the abnormal activation of microglia, synaptic loss of neurons, and changes in depressive‐like behaviors in mice. In addition, we only verified C1q and ginsenoside Rg3 in the chronic restraint stress. We also need to use the chronic unpredictable mild stress and chronic social defeated stress to verify the regulatory effect of ginsenoside Rg3 on C1q. Our research group will further explore this research direction.

## Author Contributions

Daofeng Yang: Finished the animal study and was responsible for the vitro study, wrote the manuscript. Xuerui Zhuo: Finished the animal study, was responsible for the vitro study. Hui Cai: Analyzed data and constructed the graphs. Yiming Sun: Designed the study, analyzed data and constructed the graphs, revised the manuscript.

## Ethics Statement

The animal protocols were approved by the Animal Committee of Bengbu Medical College.

## Conflicts of Interest

The authors declare no conflicts of interest.

## Data Availability

The data that support the findings of this study are available from the corresponding author upon reasonable request.
